# Thermoelectric properties of Ca_0.8_Dy_0.2_MnO_3 _synthesized by solution combustion process

**DOI:** 10.1186/1556-276X-6-548

**Published:** 2011-10-05

**Authors:** Kyeongsoon Park, Ga Won Lee

**Affiliations:** 1 Faculty of Nanotechnology and Advanced Materials Engineering, Sejong University, Seoul 143-747, Korea

**Keywords:** electrical conductivity, solution combustion process, Seebeck coefficient, power factor, Ca_0.8_Dy_0.2_MnO_3_

## Abstract

High-quality Ca_0.8_Dy_0.2_MnO_3 _nano-powders were synthesized by the solution combustion process. The size of the synthesized Ca_0.8_Dy_0.2_MnO_3 _powders was approximately 23 nm. The green pellets were sintered at 1150-1300°C at a step size of 50°C. Sintered Ca_0.8_Dy_0.2_MnO_3 _bodies crystallized in the perovskite structure with an orthorhombic symmetry. The sintering temperature did not affect the Seebeck coefficient, but significantly affected the electrical conductivity. The electrical conductivity of Ca_0.8_Dy_0.2_MnO_3 _increased with increasing temperature, indicating a semiconducting behavior. The absolute value of the Seebeck coefficient gradually increased with an increase in temperature. The highest power factor (3.7 × 10^-5 ^Wm^-1 ^K^-2 ^at 800°C) was obtained for Ca_0.8_Dy_0.2_MnO_3 _sintered at 1,250°C. In this study, we investigated the microstructure and thermoelectric properties of Ca_0.8_Dy_0.2_MnO_3_, depending on sintering temperature.

## 1. Introduction

Solid-state thermoelectric power generation based on Seebeck effects has potential applications in waste-heat recovery. Thermoelectric generation is thermodynamically similar to conventional vapor power generation or heat pumping cycles [1]. Thermoelectric devices are not complicate, have no moving parts, and use electrons as working fluid instead of physical gases or liquids [1,2]. The efficiency of thermoelectric devices is determined by the materials' dimensionless figure-of-merit, defined as *ZT *= *σα*^2^/*κT*, where *σ*, *α*, κ, and *T *are the electrical conductivity, Seebeck coefficient, thermal conductivity, and absolute temperature, respectively. To be a good thermoelectric material, it is required to have a large electrical conductivity and Seebeck coefficient as well as a low thermal conductivity. The three parameters depend on each other since they are closely related to the scattering of charge carriers and lattice vibrations. It is thus necessary to compromise among them for optimizing the thermoelectric properties [3].

Kobayashi et al. [4] proposed the possibility of (R_1-*x*_Ca_*x*_)MnO_-δ _(R: Tb, Ho, and Y) with the orthorhombic perovskite-type structure as *n*-type thermoelectric materials. Since then, the electrical transport properties of (Ca_0.9_M_0.1_)MnO_3 _(M = Y, La, Ce, Sm, In, Sn, Sb, Pb, and Bi) have been studied, and reported that partial substitution for the Ca led to a significant increase in the electrical conductivity, along with a moderate decrease in the absolute value of the Seebeck coefficient, thereby improving the dimensionless figure-of-merit [3].

It is well known that controlling the microstructure and processing, especially sintering, is a feasible route to improve the thermoelectric performance. Therefore, in this study, to improve the thermoelectric properties, nano-sized Ca_0.8_Dy_0.2_MnO_3 _powders were synthesized by the solution combustion process. The solution combustion process is favorable for synthesizing pure and nano-sized high-quality oxide powders in a short time and is cost-effective [5,6]. Subsequently, we sintered the Ca_0.8_Dy_0.2_MnO_3 _green pellets at 1150-1300°C and then investigated the microstructure and thermoelectric properties, depending on sintering temperature.

## 2. Experimental

Ca_0.8_Dy_0.2_MnO_3 _powders were synthesized by the solution combustion process. The process involved the exothermic reaction initiated by metal nitrates (oxidizer) and an organic fuel (reductant). Ca(NO_3_)_2 _· 6H_2_O, Mn(NO_3_)_2 _· 6H_2_O, Dy(NO_3_)_3 _· 5H_2_O were used as oxidizers and glutamic acid (C_5_H_9_NO_4_) as combustion fuel. The molar ratio of the metal nitrates to the fuel in the precursor solution was adjusted to be 1:1. The appropriate proportions of the metal nitrates were separately dissolved in distilled water to prepare homogeneous solutions. The glutamic acid was separately dissolved in the solutions. The resulting solution was heated slowly on a hot plate, boiled, and dehydrated, forming a highly viscous gel. Subsequently, the gel frothed and swelled with evolution of huge volume of gases. The reaction lasted for 3-4 min and produced a foam that readily crumbled into powder. The size and morphology of the resulting powders were characterized with a transmission electron microscope (TEM; JEOL JEM-2100F) operating at 200 kV. Subsequently, the synthesized powders were calcined at 900 and 1,000°C for 12 h with intermediate grinding. The calcined nanopowders were cold-pressed under 137 MPa to prepare green pellets. The pellets were sintered at 1150-1300°C at a step of 50°C in air.

The porosity of as-sintered Ca_0.8_Dy_0.2_MnO_3 _was measured by the Archimedes' principle. The crystal structure of as-sintered samples was analyzed with an X-ray diffractometer (XRD; Rigaku DMAX-2500) using Cu *K*α radiation at 40 kV and 100 mA. The microstructure of as-sintered samples was investigated with a field emission scanning electron microscope (FESEM; Hitachi S4700). To measure the thermoelectric properties as a function of temperature, the electrical conductivity *σ *and the Seebeck coefficient *α *were simultaneously measured over a temperature range of 500-800°C.

Samples for the measurements of thermoelectric properties were cut out of the sintered bodies in the form of rectangular bars of 2 × 2 × 15 mm^3 ^with a diamond saw and polished with SiC emery paper. The electrical conductivity *σ *was measured by the direct current (dc) four-probe method. For thermopower measurements, a temperature difference Δ*T *in the sample was generated by passing cool Ar gas over one end of the sample placed inside a quartz protection tube. The temperature difference Δ*T *between the two ends of each sample was controlled at 4-6°C by varying the flowing rate of Ar gas. The thermoelectric voltage Δ*E *measured as a function of the temperature difference Δ*T *gave a straight line. The Seebeck coefficient *α *was calculated from the relation *α *= Δ*E*/Δ*T*.

## 3. Results and discussion

**Figure 1:**
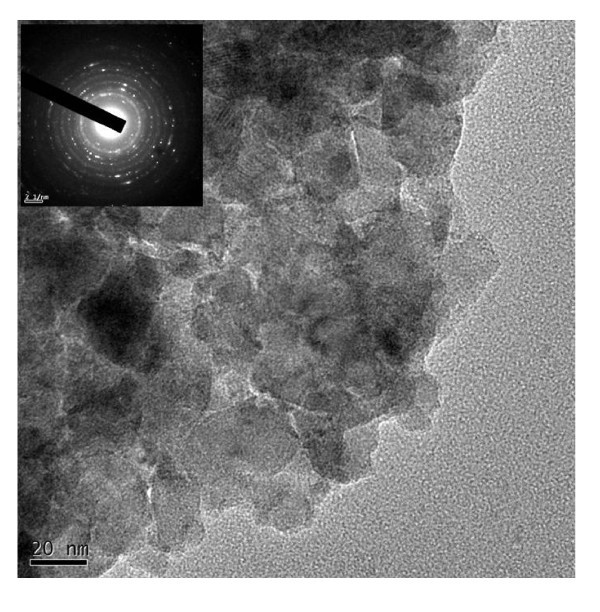
**TEM bright-field image of synthesized Ca_0.8_Dy_0.2_MnO_3 _powders**.

Figure 1 shows a TEM bright-field image of the synthesized Ca_0.8_Dy_0.2_MnO_3 _powders. The synthesized Ca_0.8_Dy_0.2_MnO_3 _powders show spherical and regular morphologies, and smooth surfaces. The average size of the synthesized powders is in nano-scale, i.e., approximately 23 nm. Obviously, this combustion processing is an extremely simple and cost-effective method for preparing Ca_0.8_Dy_0.2_MnO_3 _nanopowders, compared to conventional solid-state reaction processing.

**Figure 2:**
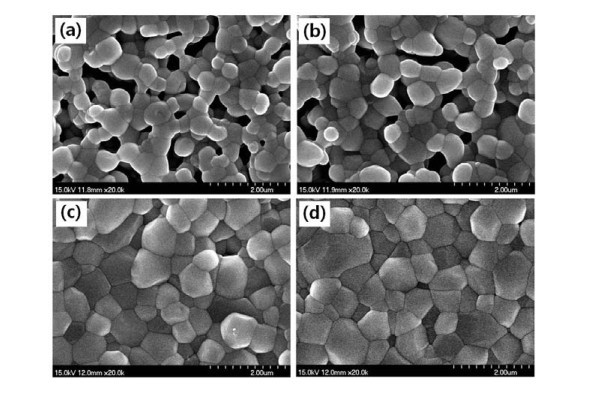
**FESEM images obtained from the surfaces of Ca_0.8_Dy_0.2_MnO_3 _sintered at (a) 1150, (b) 1200, (c) 1250, and (d) 1300°C**.

Figure 2a-d represents FESEM images obtained from the surfaces of Ca_0.8_Dy_0.2_MnO_3 _sintered at 1150, 1200, 1250, and 1300°C, respectively. Most pores are located at the grain boundaries. As the sintering temperature increases, the average grain size of the samples increases, i.e., 399, 430, 545, and 590 nm for 1150, 1200, 1250, and 1300°C, respectively. In addition, the density of the samples escalates with an increase in sintering temperature up to 1250°C, and then decreases with a further rise in sintering temperature. The densities of Ca_0.8_Dy_0.2_MnO_3 _sintered at 1150, 1200, 1250, and 1300°C are 81.5, 87.2, 98.5, and 96.3% of the theoretical density, respectively. A fine-grain size and high density are obtained even at a low sintering temperature of 1250 and 1300°C. This indicates that nano-sized powders synthesized by the glutamic acid-assisted combustion method allow for dense and fine-grained pellets at much lower sintering temperature, compared to conventional solid-state reaction processed powders. The finer powder has a larger surface energy, thus giving rise to larger densification and grain growth rates because of a high diffusivity near the surface and grain boundary during sintering [7].

**Figure 3:**
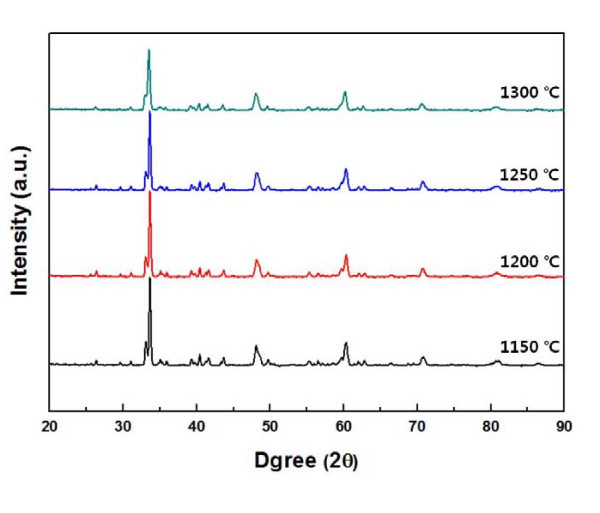
**XRD patterns of Ca_0.8_Dy_0.2_MnO_3 _sintered at various temperatures**.

The XRD patterns of the Ca_0.8_Dy_0.2_MnO_3 _sintered at various temperatures are shown in Figure 3. The sintered Ca_0.8_Dy_0.2_MnO_3 _has an orthorhombic perovskite-type structure, belonging to the *Pnma *space group [8]. The added Dy^3+ ^does not affect the crystal structure of CaMnO_3_. The crystallite size *D *of the Ca_0.8_Dy_0.2_MnO_3 _pellets can be calculated from the Scherrer formula: *D *= (0.9*λ*)/(*β*cos*θ*), where *λ *is the wavelength of radiation, *θ *is the angle of the diffraction peak, and *β *is the full width at half maximum of the diffraction peak (in radian) [9]. The calculated crystallite sizes of the sintered Ca_0.8_Dy_0.2_MnO_3 _are in the range of 20.0-24.5 nm.

**Figure 4:**
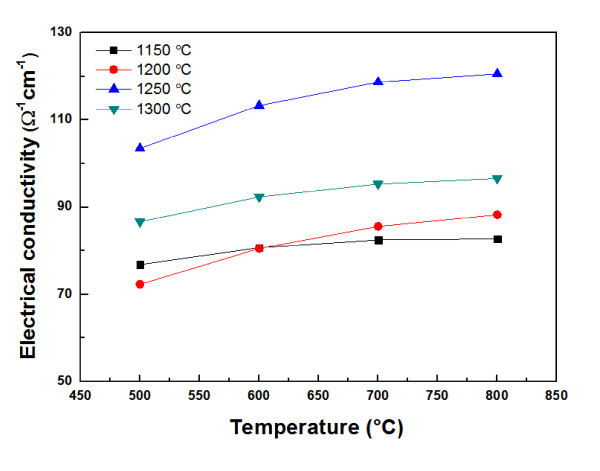
**Electrical conductivity of Ca_0.8_Dy_0.2_MnO_3 _sintered at various temperatures**.

The electrical conductivity of Ca_0.8_Dy_0.2_MnO_3 _sintered at various temperatures is shown in Figure 4. The electrical conductivity increases with increasing temperature, indicating a typical semiconducting behavior characteristic. In addition, the electrical conductivity increases with increasing sintering temperature, reaching a maximum at 1250°C, and then decreases with further increasing sintering temperature. The electrical conductivities at 800°C for the Ca_0.8_Dy_0.2_MnO_3 _samples sintered at 1150, 1200, 1250, and 1300°C are 82.8, 88.3, 120.5, and 96.6 Ω^-1 ^cm^-1^, respectively. The electrical conductivity of the Ca_0.8_Dy_0.2_MnO_3 _sintered at 1300°C is lower than that of the Ca_0.8_Dy_0.2_MnO_3 _sintered at 1250°C. This result indicates that the porosity strongly affects the electrical conductivity of Ca_0.8_Dy_0.2_MnO_3_. Pores act as scattering centers for conduction, decreasing the time between electron scattering events of charge carriers. The highest electrical conductivity (120.5 Ω^-1 ^cm^-1^) is obtained for the Ca_0.8_Dy_0.2_MnO_3 _sintered at 1250°C.

**Figure 5:**
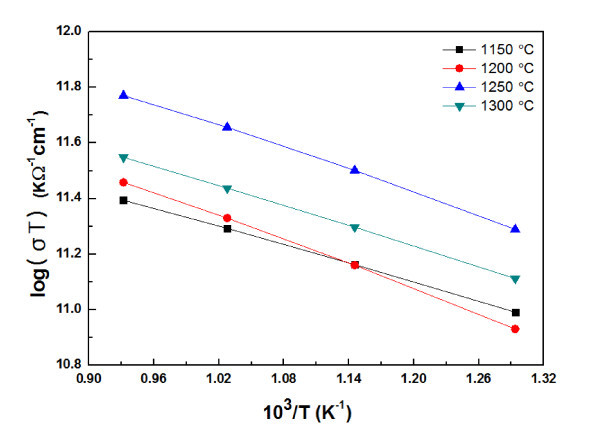
**A relationship between the log(*σT*) and 1000/*T *for Ca_0.8_Dy_0.2_MnO_3 _as a function of sintering temperature**.

A relationship between the log(*σT*) and 1000/*T *for Ca_0.8_Dy_0.2_MnO_3 _as a function of sintering temperature is shown in Figure 5. We can find a nearly linear relationship between log(*σT*) and 1000/*T *over the measured temperature range. The activation energy (*E*_a_) for conduction at high temperatures (500-800°C) is calculated from the slope of the log(*σT*) and 1000/*T*. The calculated activation energies of the Ca_0.8_Dy_0.2_MnO_3 _sintered at 1150, 1200, 1250, and 1300°C are 0.096, 0.126, 0.115, and 0.104 eV, respectively. This means that the conduction of these samples is caused by a thermally activated small polaron hopping [10]. A small polaron is formed when the effective mass of the rigid lattice hole is large and coupling to optical phonons is strong [11].

In the polaron hopping conduction, an electron moves by a thermally activated hopping process from one localized state to another with the activation energy *E*_h _[12]. The electrical conductivity *σ *is written as *σ *= (*C*/*T*)exp(-*E*_h_/*k*_B_*T*), where *C*, *T*, *E*_h_, and *k*_B _are the charge carrier concentration, the absolute temperature, the activation energy, and the Boltzmann constant, respectively [3]. The electrical conductivity of the small polaron hopping conduction in the adiabatic case is given as *σ *= *neμ *= *nea*^2^(*A*/*T*)exp(-*E*_h_/*k*_B_*T*), where *n *is the carrier concentration, *e *is the electrical charge of the carrier, *μ *is the carrier mobility, *a *is the intersite distance of hopping, *E*_h _is the activation energy for hopping, and *A *is the pre-exponential tern related to the carrier scattering mechanism, respectively [3,13].

**Figure 6:**
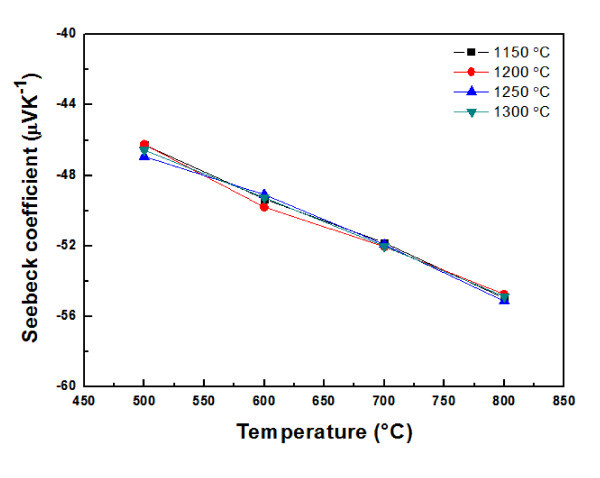
**Seebeck coefficient of Ca_0.8_Dy_0.2_MnO_3 _as a function of temperature**.

The Seebeck coefficient of Ca_0.8_Dy_0.2_MnO_3 _as a function of temperature is shown in Figure 6, depending on sintering temperature. The absolute value of the Seebeck coefficient for Ca_0.8_Dy_0.2_MnO_3 _gradually increases with an increase in temperature. The sign of the Seebeck coefficient is negative over the measured temperature range, indicating *n*-type conduction. The absolute values of the Seebeck coefficients at 800°C for the Ca_0.8_Dy_0.2_MnO_3 _sintered at 1150, 1200, 1250, and 1300°C are 55.0, 54.7, 55.1, and 54.9 μV K^-1^, respectively, indicating sintering temperature has no significant influence on the Seebeck coefficient.

**Figure 7:**
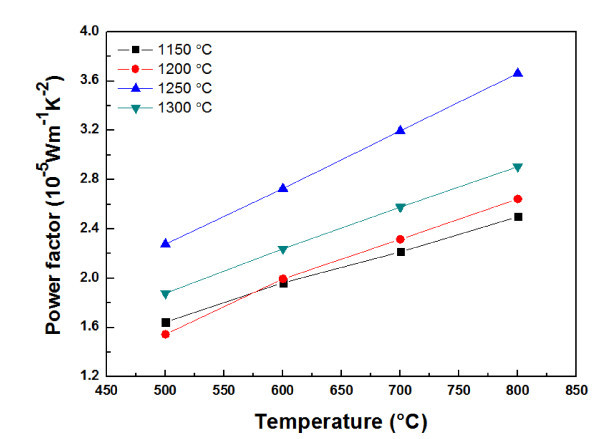
**Power factor of Ca_0.8_Dy_0.2_MnO_3 _sintered at various temperatures**.

The power factor *σα*^2 ^is calculated using the electrical conductivity *σ *and the Seebeck coefficient *α*. The power factor obtained from the data in Figures 4 and 6 is plotted in Figure 7. At a given sintering temperature, the power factor increases with an increase in temperature. In addition, the power factor increases with sintering temperature up to 1250°C and then decreases for higher sintering temperature. The highest power factor (3.7 × 10^-5 ^Wm^-1 ^K^-2 ^at 800°C) is obtained for the Ca_0.8_Dy_0.2_MnO_3 _sintered at 1250°C. From the above results, it is believed that controlling the sintering temperature of Ca_0.8_Dy_0.2_MnO_3 _is important for improving its thermoelectric properties.

## 4. Conclusion

We synthesized Ca_0.8_Dy_0.2_MnO_3 _nanopowders (approximately 23 nm in size), which showed spherical and regular morphologies, and smooth surfaces, by the glutamic acid-assisted combustion method. The nano-sized powders led to dense and fine-grained pellets at low sintering temperature. The average grain sizes of the Ca_0.8_Dy_0.2_MnO_3 _sintered at 1150, 1200, 1250, and 1300°C were 399, 430, 545, and 590 nm, respectively. In addition, the densities of the Ca_0.8_Dy_0.2_MnO_3 _sintered at 1150, 1200, 1250, and 1300°C were 81.5, 87.2, 98.5, and 96.3% of the theoretical density, respectively. The Ca_0.8_Dy_0.2_MnO_3 _sintered had an orthorhombic perovskite-type structure, belonging to the *Pnma *space group. The electrical conductivity increased with increasing sintering temperature, reaching a maximum at 1250°C, and then decreased with further increasing sintering temperature. However, a noticeable change in the Seebeck coefficient of Ca_0.8_Dy_0.2_MnO_3 _sintered at various temperatures was not evident. The Ca_0.8_Dy_0.2_MnO_3 _sintered at 1250°C showed the highest power factor (3.7 × 10^-5 ^Wm^-1 ^K^-2^) at 800°C. It is necessary to control the sintering temperature of Ca_0.8_Dy_0.2_MnO_3 _for improving the thermoelectric properties.

## Competing interests

The authors declare that they have no competing interests.

## Authors' contributions

KP conceived of the study, participated in its design and coordination, and drafted the manuscript. GWL carried out the synthesis, microstructure analysis, and thermoelectric studies. All authors read and approved the final manuscript.
